# Analysis of Mechanical Properties and Structural Analysis According to the Multi-Layered Structure of Polyethylene-Based Self-Reinforced Composites

**DOI:** 10.3390/polym15204055

**Published:** 2023-10-11

**Authors:** Seonghun Yu, Junhee Lee, Jongkyu Kim, Hojong Chang, Chansol Kang, Jeehyun Sim

**Affiliations:** 1 DYETEC (Dyeing & Finishing Technology Institute), Computer Aided Engineering (CAE) Center, Daegu 41706, Republic of Korea; enviro1234@dyetec.or.kr (S.Y.);; 2 Department of Energy Engineering, Shinhan University, 95, Hoam-ro, Uijeongbu-si 11644, Republic of Korea; 3 KAIST Institute for Information Technology Convergence Intergrated Sensor Team, KAIST, Daejeon 34141, Republic of Korea; 4 Department of Advanced Materials Engineering, Shinhan University, 95, Hoam-ro, Uijeongbu-si 11644, Republic of Korea

**Keywords:** polyethylene-based self-reinforcing composites, hot stamping, recyclability, interfacial bonding force, compression simulation

## Abstract

In this research, a self-reinforced composite material was manufactured using a single polyethylene material, and this self-reinforced composite material has excellent recyclability and is environmentally friendly compared to composite materials composed of other types of material, such as glass fiber reinforced composites (GFRP) and carbon fiber reinforced composites (CFRP). In this research, the manufactured self-reinforced composite material consists of an outer layer and an inner layer. To manufacture the outer layer, low density polyethylene (LDPE) films were laminated on high density polyethylene (HDPE) fabrics and knitted fabrics, and composite materials were prepared at various temperatures using hot stamping. A 3D printing process was utilized to manufacture the inner layer. After designing a structure with a cross-sectional shape of a triangle, circle, or hexagon, the inner layer structure was manufactured by 3D printing high-density polyethylene material. As an adhesive film for bonding the outer layer and the inner layer, a polyethylene-based self-reinforced composite material was prepared using a low-density polyethylene material. Input data for simulation of self-reinforced composite materials were obtained through tensile property analysis using a universal testing machine (UTM, Shimadzu, Kyoto, Japan), and the physical property values derived as output data and actual experimental values were obtained. As a result of the comparison, the error rate between simulation data and experimental data was 5.4% when the shape of the inner layer of self-reinforced composite material was a hexagon, 3.6% when it was a circle, and 7.8% when a triangular shape showed the highest value. Simulation in a virtual space can reduce the time and cost required for actual research and can be important data for producing high-quality products.

## 1. Introduction

Self-reinforced composite (SRC) is a composite material manufactured by melting and recrystallizing the matrix at an appropriate temperature and pressure with the same polymeric reinforcement and matrix [1,2,3,4,5,6]. SRC is a composite material manufactured using the same polymer, and has excellent recyclability compared to composite materials composed of other types. It has the advantage of excellent compatibility [7,8,9,10,11]. in the research of Chandran and Padmanabhan, the interface of the self-reinforced composite material was observed, and it was confirmed that the interfacial bonding force was higher than that of the existing composite material, which is a heterogeneous material [12]. Accordingly, it is a product that can replace existing glass fiber reinforced plastics (GFRP) and carbon fiber reinforced plastics (CFRP) in some industrial fields [13,14,15,16,17,18].

As a study for manufacturing self-reinforced composite materials, Loos et al. developed a new technology to produce SRC using bi-component tape material [19]. In this research, a copolymer with a lower melting point than bi-component tape material was used as a matrix, and methods for manufacturing SRC included a powder impregnation method, solvent impregnation method, and film lamination method. The solution impregnation method has good impregnability because the resin is dissolved in a solvent, but it has the fatal disadvantage of poor productivity because the solvent volatilization time is long [20,21]. On the other hand, the film lamination method is a method that is widely used due to a simple manufacturing process. Since it is manufactured with a relatively simple process, it has excellent productivity and has the advantage that the physical properties of the manufactured product are uniform [22,23,24,25,26]. Santos has conducted research on several types of self-reinforced composite materials manufactured using a single polymer such as polypropylene, polyester, and polyamide [27]. The tensile strength, flexural strength, and impact strength of self-reinforced composites using a single material were analyzed. However, no research has been conducted on self-reinforced composite materials using polyethylene in any research.

Therefore, in this study, a polyethylene material-based self-reinforced composite material was manufactured using the film lamination method, and a physical property database was established according to the manufacturing process conditions. Through this, we tried to secure the reliability of the simulation value. The material property database was used as input data for simulation, and output data, which are the result of the simulation, were also derived. The self-reinforced composite material manufactured in this study consists of an outer layer and an inner layer. To manufacture the outer layer, low density polyethylene (LDPE) films were laminated on high density polyethylene (HDPE) fabrics and knitted fabrics, and composite materials were prepared at various temperatures using hot stamping. A 3D printing process was utilized to manufacture the inner layer [28,29,30]. First, after designing a structure with a cross-sectional shape of a triangle, circle, or hexagon, the inner layer structure was manufactured by 3D printing high-density polyethylene material. An inner layer with a thickness of 20 mm was manufactured using HDPE plastic for 3D printing. As an adhesive film for bonding the outer layer and the inner layer, a polyethylene-based self-reinforced composite material was prepared using a low-density polyethylene material. Input data for the simulation of self-reinforced composite materials were obtained through tensile property analysis using a Universal Testing Machine (UTM, Shimadzu, Kyoto Japan), and the physical property values derived as output data and actual experimental values were also obtained by proceeding with comparison, and we tried to confirm the reliability of the simulation values, which are the output data. We tried to secure the reliability of simulation values by comparing the similarity between experimental and simulated values, and reliable simulation data are innovative because they can reduce the time and cost required for research. The software used for 3D modeling was ABAQUS CAE (Simulia, version 6.6, New York, NY, USA). The unit cell was defined using two-dimensional beam elements, and the effective elastic modulus and Poisson’s ratio in each direction were derived. The simulation values of self-reinforced composite materials have meaningful results. By predicting the physical properties of self-reinforced composite materials through simulation, the cost and time required for product development can be reduced. This is because physical properties can be known without actually producing a sample. Composite materials used in the mobility industry or aerospace industry take a lot of time and money to manufacture. The technology of this study can be considered groundbreaking because the performance of these composite materials can be known in advance through simulation.

## 2. Materials and Methods

### 2.1. Materials and Manufacture of Composite Materials

**Figure 1:**
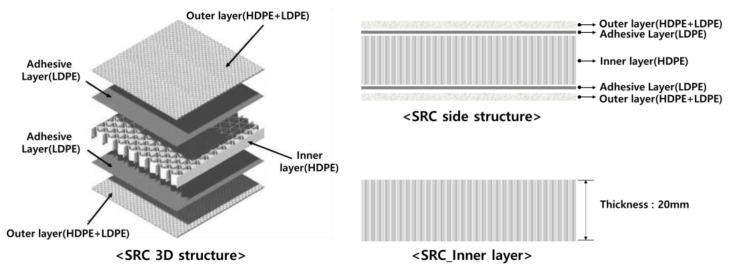
Self-reinforced composite structure (outer layer and inner layer).

The structure of the self-reinforced composite material to be manufactured is shown in Figure 1, and the self-reinforced composite material is composed of an outer layer and an inner layer. The outer layer of the self-reinforced composite material was prepared using HDPE fabric (woven, knitted) and LDPE film. The structures of HDPE fabrics and knitted fabrics are shown in Table 1. HDPE fabric (Kolon industry, Ulsan-si, Republic of Korea) used a total of three types of fabrics including plain weave, twill weave, and satin weave. Two types of HDPE knitted fabrics (Kolon industry, Ulsan-si, Republic of Korea) were used: circular knitting and warp knitting. The types and basic information of HDPE fabrics are shown in Table 1.

The LDPE film (SK chemical, Seongnam-si, Republic of Korea) used a material with a thickness of 0.03 mm and a melt index (M.I) of 3.1 g/10 min. The thickness of the HDPE fabric and knitted fabric was the same at 0.3 mm. HDPE fabric was set to 5 ply and LDPE film was set to 15 ply to produce an outer layer of self-reinforcing composite material composed of HDPE fabric and LDPE film. An outer layer of material was prepared. The film content of the outer layer of the self-reinforced composite material was derived using Equation (1)

(1) $$R/C=\frac{R}{F+R}\times 100$$

In which,

R/C: LDPE film (resin) content, %;
R: Resin weight, kgf;
F: Fabric weight, kgf.

**Figure 2:**
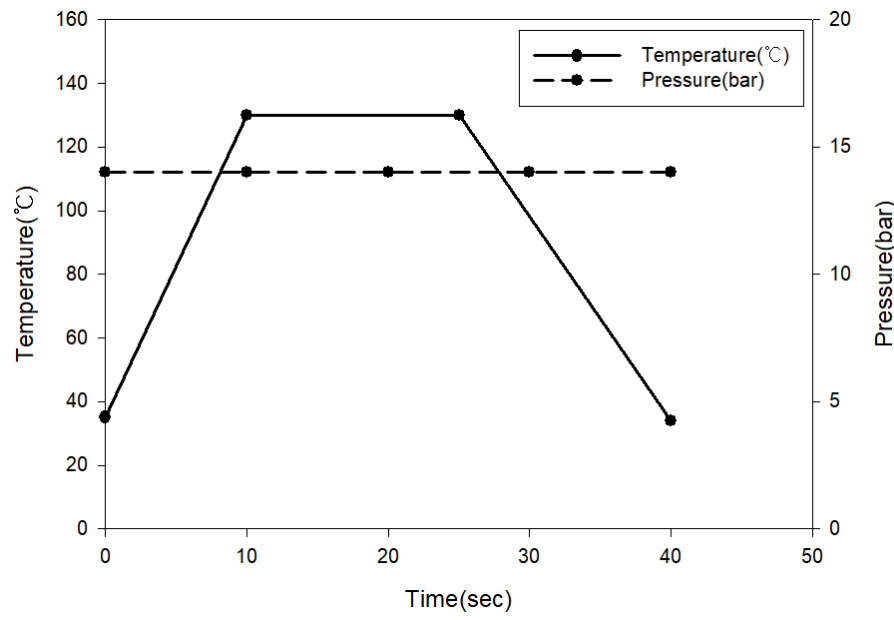
Temperature and pressure conditions for manufacturing outer layer of self-reinforced composites.

The melting point of the HDPE material is 143.6 °C and the melting point of LDPE material is 117.4 °C. Accordingly, the temperature and pressure conditions for manufacturing the outer layer of the self-reinforced composite material are shown in Figure 2.

In the case of the inner layer of the self-reinforced composite material, an inner layer with a thickness of 20 mm was prepared using HDPE plastic (SK chemical, Seongnam-si, Republic of Korea) for 3D printing, and the cross-sectional shape was set to triangle, circle, and hexagon. The designed shape is shown in Table 2, and 2-ply LDPE films were laminated on the upper and lower sides of the inner layer of the self-reinforced composite material, respectively, to combine the inner and outer layers. Since the HDPE material should not melt, the molding conditions for bonding the outer and inner layers of the self-reinforced composite material were carried out in the same way as in Figure 2.

### 2.2. Thermal Characterization

The thermal characteristics of the outer layer of the manufactured self-reinforced composite material were analyzed according to the manufacturing conditions to analyze the correlation between the constituent materials and structures constituting the outer layer and the thermal characteristics. To analyze the thermal characteristics, the thermal conductivity was derived according to the manufacturing conditions of the outer layer of the self-reinforced composite material, and the thermal conductivity was calculated according to Equation (2).

(2) $$Thermal\;Condctivity=\frac{W\times D}{A\times ∆T}$$

In which,

W: Heat flow, J/s;
D: Thickness of specimen, m;
A: Heating plate area, m^2^;
ΔT: Temperature difference between the specimen stand and the hot plate, K.

Self-reinforced composite materials can be applied to various industrial fields depending on the characteristics of the material used. Products with high thermal conductivity can be used for the purpose of releasing heat, and products with low thermal conductivity can be used for insulation purposes.

### 2.3. Mechanical Characterization

The evaluation of mechanical properties such as tensile strength, flexural strength, and shear strength was conducted only for the outer layer of the manufactured self-reinforced composite material, and simulation was conducted based on the established data. In the case of the tensile strength of the outer layer of the self-reinforced composite material, the test was conducted with a universal testing machine (UTM, Shimadzu, Kyoto, Japan) in accordance with ASTM D3039 [31]. The size of the specimen was 175 × 25 mm^2^. Tensile strength and tensile modulus were measured at a tensile speed of 2 mm/min, five specimens were measured, and the average value was obtained. In the case of flexural strength, it was conducted in accordance with ASTM D790 [32], the thickness of the specimen and the length of the support span were set at a ratio of 16:1, and the flexural test was conducted. In the case of shear strength, after preparing a specimen in the form of a v-notch in accordance with ASTM D5379 [33], the test was conducted with a universal tensile tester. The size of the specimen was 80 × 10 mm^2^, and the shear strength was measured at the speed of the experiment at 2 mm/min. Five specimens were measured and the average value was obtained. Young’s modulus and Poisson’s ratio value for the HDPE material used to manufacture the inner layer of the self-reinforced composite material were provided by the material supplier (SK Chemicals, Seongnam-si, Republic of Korea).

The mechanical property evaluation data of the outer layer of the self-reinforced composite material and the material property data of the inner layer were used as input data for the simulation. The compression test was conducted with a universal testing machine by setting the compression speed to 2 mm/min.

### 2.4. 3D Modeling and Boundary/Load Condition

Based on the tensile property evaluation data of the outer layer of the self-reinforced composite material and the material data used to manufacture the inner layer of the self-reinforced composite material, the composite material was 3D modeled in a virtual space, and the external compressive load of the self-reinforced composite material in which the inner and outer layers were combined. After setting the boundary conditions, we tried to conduct a physical property prediction simulation according to the external load condition and the inner layer structure (hexagon, circle, triangle) of the self-reinforced composite material. For the input data of the outer layer of self-reinforced composite material, the resultant value of specimen #3 was used, and a simulation was conducted according to the structure of the inner layer of the self-reinforced composite material.

The software used for 3D modeling was ABAQUS CAE (Simulia, version 6.6, New York, NY, USA). A unit cell was defined using a two-dimensional order element, and the effective elastic modulus in each direction and the O′ distribution were obtained. The size or shape of the element was not significantly affected and the unusable isometric site was used. The element type was assumed to be C3D8R (An 8-node linear brick, reduce integration, hourglass control). The external consumption condition was set at a compression speed of 2 mm/min in the vertical direction on top of the self-reinforced composite material combined with the inner and outer layers. The boundary conditions were simulated by completely fixing the side and bottom surfaces of the self-defense reinforced composite material.

## 3. Results and Discussion

### 3.1. Thermal Characterization Results

Table 3 shows the thermal conductivity analysis results, which are the thermal characteristics of the outer layer of the manufactured self-reinforced composite material according to the manufacturing conditions. HDPE thermal conductivity values vary depending on factors such as the thickness and density of the fabric, and since the thermal conductivity of the air layer is 0.024 W/m·k, the thermal conductivity value varies depending on the content of the air layer [34,35,36]. The HDPE woven fabrics manufactured in this study have different structures such as plain weave, twill weave, satin weave, warp weave, and circular weave. In the case of the outer layer of the self-reinforced composite material made of HDPE fabric, the value increased by about 25.2% or more compared to the average value of the outer layer of the self-reinforced composite material to which the HDPE knitted fabric was applied. This is considered to be because the outer layer of the self-reinforced composite made of HDPE knitted fabric has a wider gap between materials and has a relatively larger number of air layers compared to the outer layer of the self-reinforced composite made of HDPE fabric.

On the other hand, the error rate between HDPE fabrics (plain, twill, satin) showed an approximate value within ±5%, and the thermal conductivity value of the outer layer of the self-reinforced composite made of HDPE satin weave was 7.1% higher than that of the self-reinforced composite made of HDPE plain weave, which showed a higher value. In the case of the outer layer of the self-reinforced composite material made of satin weave, it is judged that the thermal conductivity is high because the gap between materials is tighter than that of plain weave, and the air layer content is relatively small due to the high density of the woven fabric [37,38,39]. Heat transfer by convection can be calculated according to Formula (3), and the surface area where convection heat transfer occurs is relatively larger in the HDPE fabric, and among the fabrics, the satin weave has the largest surface area, so it is judged that the thermal conductivity is high.

(3) $$Q_{conv}=hA_{s}\left(T_{s}-T_{oo}\right)[W]$$

In which,

*h*: Convective heat transfer coefficient, W/m^2^·K;
*A_s_*: Surface area where convective heat transfer occurs, m^2^;
*T_s_*: Solid surface temperature, K;
*T_oo_*: The temperature of a fluid that is not affected by the temperature of a solid, K.

### 3.2. Mechanical Characterization Result

**Figure 3:**
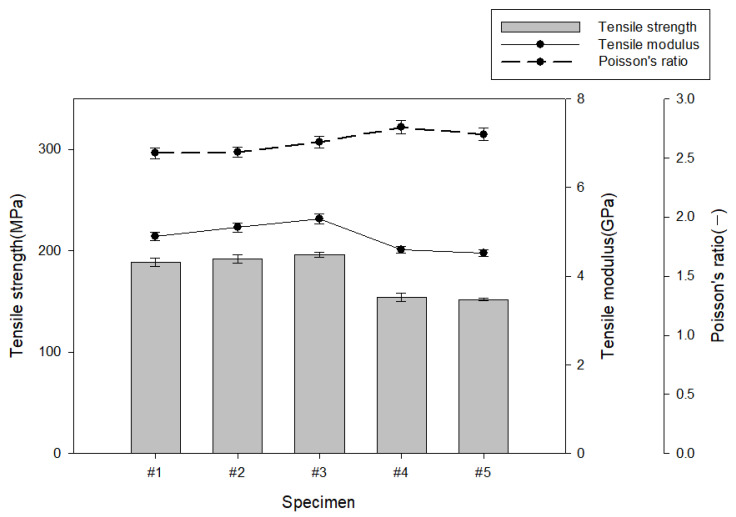
Tensile characteristic analysis results for each manufacturing condition of the outer layer of self-reinforced composites.

The film content calculated according to Equation (1) is shown in Table 4. Figure 3 shows the measurement results of tensile strength, tensile modulus, and Poisson’s ratio of the HDPE/LDPE self-reinforced composites. As a result of analyzing the film content for each manufacturing condition of the outer layer of the self-reinforced composite material, the average value of the HDPE knitted fabric was improved by more than 11.5% compared to the average value of the fabric. This is because, when preparing a specimen for mechanical property analysis, the specimen must be manufactured with a constant thickness, so the fabric having a relatively high reinforcing material content per unit area was measured to have a low reinforcing material content.

In the case of the result of analyzing the tensile properties of the outer layer of the self-reinforced composite material, the #3 specimen showed 196 MPa, a value improved by more than 28.9% in tensile strength compared to the #5 specimen. In the case of polyethylene-based self-reinforcing composites, since they are manufactured by applying the same polyethylene material, the thermal expansion coefficient between the base material and the reinforcing material is basically the same, so the interfacial bonding strength is excellent, but the tensile properties are also different because the structure of the reinforcing material is different. It is believed that the tensile strength and tensile modulus of elasticity are improved because the HDPE fabric has a denser structure compared to the HDPE knitted fabric and has a higher ratio of reinforcing materials oriented in the tensile direction. On the other hand, the poisson’s ratio value of the #3 specimen showed a value that improved by more than 3.7% compared to the #1 specimen, which is because the #3 specimen has relatively more intersections of warp and weft yarns in terms of fabric structure, so the frictional force is improved compared to the #1 specimen, and the tensile strength and tensile elasticity are judged to be such that that the coefficient increased slightly.

**Figure 4:**
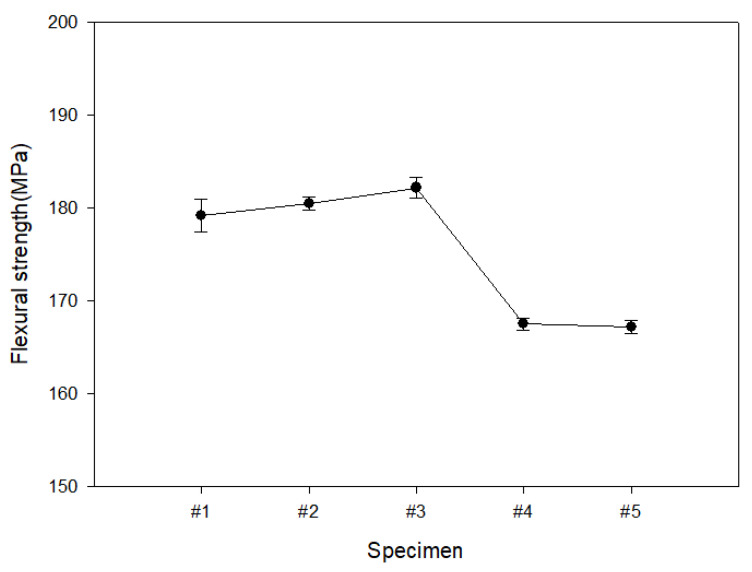
Results of analysis of the flexural characteristics of the outer layer of self-reinforced composite materials according to manufacturing conditions.

Figure 4 is the result of analyzing the flexural strength of the outer layer of the self-reinforced composite material. Similar to the tensile strength analysis result, the #3 sample woven with satin weave showed the highest value at 182.2 MPa, but the difference between the maximum and minimum values of the measured values was 8.2%, which was different from the tensile strength analysis results. When a bending load is applied in the vertical direction, the internal resistance of the #3 specimen, which has a relatively dense structure, is high, but the content of the reinforcing material is relatively low compared to the knitted fabric. Judging from these results, it is judged that the flexural strength of the outer layer of the self-reinforced composite material is influenced by the content of the base material as well as the structure of the reinforcing material. Flexural strength of the outer layer of the self-reinforced composite material shows significant results. Specimen #3 showed the highest measured flexural strength. This means that specimen #3 has a dense structure. Materials with high flexural strength are likely to be used in industrial products or structures that must withstand external pressure or load. However, because the absolute value is not high, there is a need to continuously conduct research and development to improve flexural strength.

**Figure 5:**
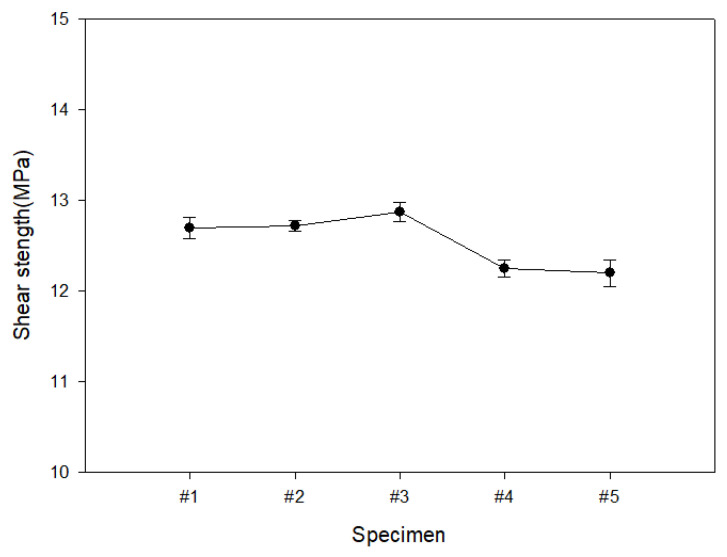
Results of analysis of shear characteristics for outer layer of self-reinforced composite material by manufacturing conditions.

Figure 5 is the shear strength analysis result of the outer layer of the self-reinforced composite material. The shear strength tends to be higher as the interfacial bonding force of the specimen is better, and the shear strength results showed an approximate value within ±5% for all specimens #1 to #5. Self-reinforced composites were manufactured using composite materials of the same series. Because the self-reinforced composite material was manufactured using the same series of composite materials, the thermal expansion coefficient is almost the same. Therefore, it is judged that the shear strength values were almost similar.

In the case of carbon composite materials manufactured using carbon fiber and thermosetting epoxy resin, the shear strength value decreases because the thermal expansion coefficients of carbon fiber and thermosetting epoxy resin are different [40,41]. On the other hand, because self-reinforced composites have excellent shear strength values, they can be used in various industrial fields by replacing carbon composite materials. If we continue to research self-reinforced composite materials, we can produce better results than the current self-reinforced composite materials.

### 3.3. Simulation Results

Table 5 shows the input data generation results of the outer and inner layers of the self-reinforced composite material for the prediction simulation of physical properties. As the input data, the result value of the #3 specimen derived from the analysis of tensile properties and the data value of the material used for manufacturing the inner layer of the self-reinforced composite material were used.

Simulation results for the compressive load values according to the inner layer structure (triangle, circle, hexagon) of the self-reinforced composite material were derived, and the results of generating the 3D model of the inner layer structure of the self-reinforced composite material are shown in Table 6.

**Figure 6:**
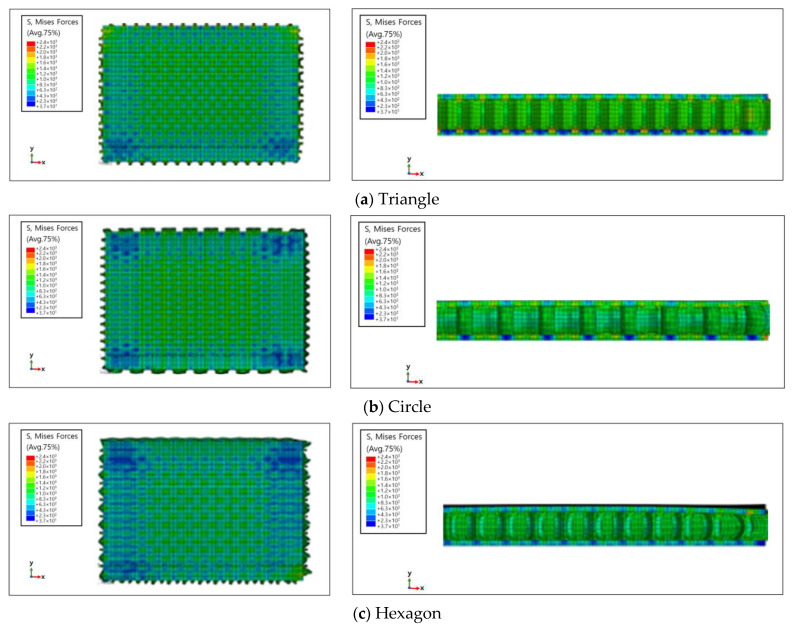
Structural analysis results according to the inner layer structure of self-reinforced composite material.

The structural analysis results according to the inner layer structure of the self-reinforced composite material in which the outer and inner layers are combined are shown in Figure 6, and the comparison results between the actual compressive load test value and the simulation result value according to the inner layer structure are shown in Table 7.

The error rate between the simulation data and simulation data was 5.4% when the shape of the inner layer of the self-reinforced composite material was a hexagon, 3.6% when it was a circle, and 7.8% when it was a triangle, showing the highest value. The reason for the error between the actual compression test value and the simulation data due to the damage caused by thermal deformation during the molding process for manufacturing the outer layer of self-reinforced composite material and the occurrence of unlaminated parts during the 3D printing process for manufacturing the inner layer of self-reinforced composite material are judged [42,43,44,45,46].

## 4. Conclusions

In this study, after manufacturing a polyethylene material-based self-reinforced composite material, a physical property database was established according to manufacturing process conditions, and through a physical property database-based property prediction simulation, the reliability of the simulation value was secured by comparing the experimental value with the simulated value. The conclusion was as follows.

The self-reinforced composite material consists of an outer layer and an inner layer, and the outer layer of the self-reinforced composite material is composed of a woven fabric and a film layer. As a result of analyzing the thermal characteristics, the value increased by about 25.2% or more than the average value of the outer layer of the self-reinforced composite material to which the HDPE fabric was applied.

As a result of analyzing the mechanical properties of the outer layer of the self-reinforced composite material, the tensile value and the flexural strength value of the specimen made of HDPE fabric showed a maximum improvement of 28.9%. In the case of shear strength, an approximate value within ±5% was shown. Because they are manufactured by applying the same polyethylene material, the thermal expansion coefficients between the base material and the reinforcing material are basically the same, so the interfacial bonding strength is excellent, but it is judged that the tensile properties are also different because the structure of the reinforcing material is different.

As a result of analyzing the compressive strength of the specimen in which the outer and inner layers of the self-reinforced composite material were combined, the error rate between simulation data and simulation data was 5.4% when the shape of the inner layer of the self-reinforced composite material was a hexagon and 3.6% when the shape was a circle. The triangle showed the highest value at 7.8%. The reason for the error between the compression test value and the simulation data seems to be due to the damage mechanism due to thermal deformation generated during the molding process.

In this research, the thermal and mechanical properties of the self-reinforced composite material based on polyethylene were analyzed, and a correlation analysis between the experimental and simulated values was conducted through the simulation based on the physical property database. In the future, it is expected that various guidelines will be presented by changing the material and manufacturing process conditions according to the application field.

## Figures and Tables

**Table 1:** HDPE fabric information for manufacturing outer layer of self-reinforced composites.

Sample		Denier (−)	Weight (g/m^2^)	Thickness (mm)	Structure
#1	Plain	200	54.8	0.54	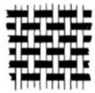
#2	Twill	200	55.0	0.55	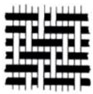
#3	Satin	200	56.2	0.54	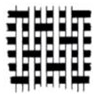
#4	Warp knitting	200	52.1	0.54	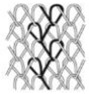
#5	Weft knitting	200	52.0	0.55	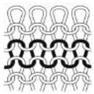

**Table 2:** Type of self-reinforced composite inner layer.

Sample		Mimetic Diagram	
Inner layer	Hexagon	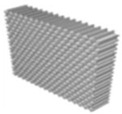	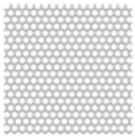
	Circle	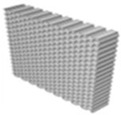	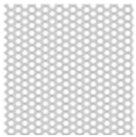
	Triangle	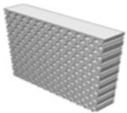	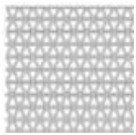

**Table 3:** Result of analyzing the thermal properties of the outer layer of self-reinforced composites.

Sample		Thermal Conductivity (W/m·k)	Specimen
#1	Plain	0.331	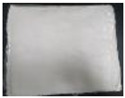
#2	Twill	0.333	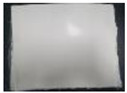
#3	Satin	0.346	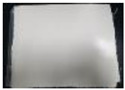
#4	Warp knitting	0.441	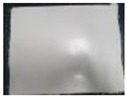
#5	Weft knitting	0.443	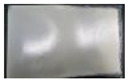

**Table 4:** Result of analysis of reinforcing material content for each manufacturing condition of the outer layer of the self-reinforced composite material.

Sample	#1	#2	#3	#4	#5
Result	39.67 (±0.03)	39.22 (±0.04)	38.73 (±0.02)	42.61 (±0.08)	42.82 (±0.09)

**Table 5:** Result of deriving input data for prediction simulation of self-reinforced composite material properties.

Materials	Input Data	
	Young’s Modulus (GPa)	Poisson’s Ratio
Self-Reinforced Composite Outer Layer	5.21 (±0.07)	0.25 (±0.02)
Self-Reinforced Composite inner Layer	5.14 (±0.05)	0.26 (±0.01)

**Table 6:** 3D modeling result of inner layer of self-reinforced composite material.

Specimen	Triangle	Circle	Hexagon
3D modeling of Outer Layer	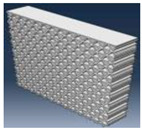	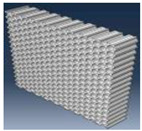	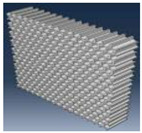
	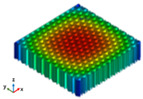	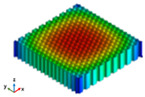	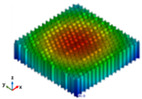

**Table 7:** Comparison of actual experimental values and simulation results according to self-reinforced composite materials.

-	Experment	Simulation	Error Rate (%)
	Max. Compressive Load (N) (at 2 mm)	Max. Compressive Load (N) (at 2 mm)	
Hexagon	4027.5	4244.9	5.4
Circle	10,857.7	11,248.5	3.6
Triangle	8765.2	9448.8	7.8
graph	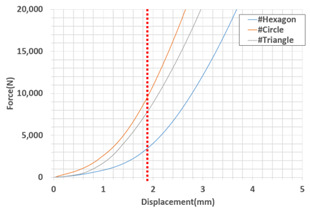	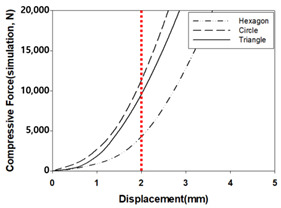	-

## Data Availability

The data presented in this study are available from the corresponding author upon reasonable request.
